# Generation of Well-Defined Micro/Nanoparticles via Advanced Manufacturing Techniques for Therapeutic Delivery

**DOI:** 10.3390/ma11040623

**Published:** 2018-04-18

**Authors:** Peipei Zhang, Junfei Xia, Sida Luo

**Affiliations:** 1 Department of Material Processing and Controlling, School of Mechanical Engineering & Automation, Beihang University, Beijing 100191, China; 2 Department of Bioengineering, Northeastern University, Boston, MA 02115, USA; j.xia@northeastern.edu

**Keywords:** advanced manufacturing techniques, micro/nanoparticles, controllable features, cells, in vivo, therapeutic delivery

## Abstract

Micro/nanoparticles have great potentials in biomedical applications, especially for drug delivery. Existing studies identified that major micro/nanoparticle features including size, shape, surface property and component materials play vital roles in their in vitro and in vivo applications. However, a demanding challenge is that most conventional particle synthesis techniques such as emulsion can only generate micro/nanoparticles with a very limited number of shapes (i.e., spherical or rod shapes) and have very loose control in terms of particle sizes. We reviewed the advanced manufacturing techniques for producing micro/nanoparticles with precisely defined characteristics, emphasizing the use of these well-controlled micro/nanoparticles for drug delivery applications. Additionally, to illustrate the vital roles of particle features in therapeutic delivery, we also discussed how the above-mentioned micro/nanoparticle features impact in vitro and in vivo applications. Through this review, we highlighted the unique opportunities in generating controllable particles via advanced manufacturing techniques and the great potential of using these micro/nanoparticles for therapeutic delivery.

## 1. Introduction

**Figure 1:**
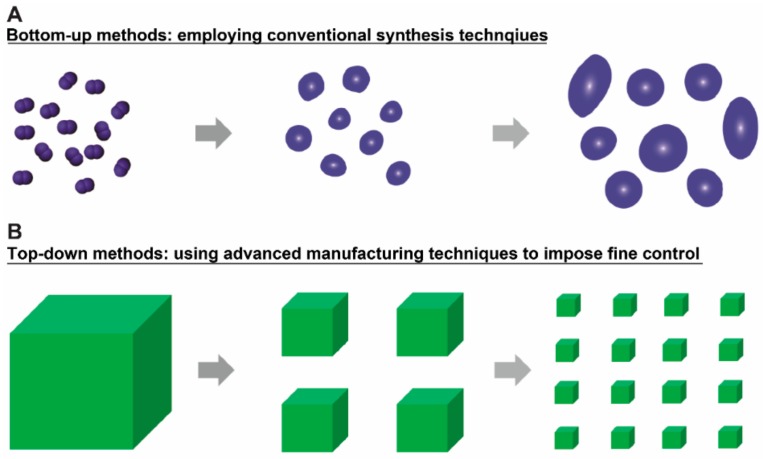
The existing micro/nanoparticle production techniques can be classified into two methods: (**A**) Bottom-up methods. The bottom-up methods employ conventional synthesis techniques (i.e., emulsion) to generate micro/nanoparticles with several shapes, including spherical, rod, etc. The particles usually vary in sizes and are not controllable in other properties such as cargo loading; (**B**) Top-down methods. This group of methods relies on using advanced manufacturing techniques to impose fine spatial control over the materials. Due to this spatial control, the micro/nanoparticles from these methods have well-controlled size, shape, surface property and component materials. This review focused on introducing micro/nanoparticles produced by advanced manufacturing techniques and discussing the applications of these micro/nanoparticles in delivering therapeutic agents.

Micro/nanotechnologies are broadly employed in biomedical applications, especially in the drug delivery field [1,2,3]. Generally, the techniques for creating micro/nanoparticles can be classified into two groups, namely the bottom-up and top-down methods (Figure 1A,B). Most conventional techniques such as double-emulsion and sol-gel are grouped under the bottom-up methods, through which the materials at micro or nano scales can be assembled from the scale of chemical molecules [4]. The relevant techniques can be used for producing a large quantity of micro/nanoparticles with several shapes, including spherical, rod, etc. (Figure 1A) [5,6]. For biomedical applications, micro/nanoparticles generated from these techniques have been widely explored for varying biomedical applications, including drug delivery [7,8]. Despite the broad applications, studies require the development of micro/nanoparticles with better control in terms of their features (i.e., size, shape, cargo loading level, surface property, etc.)—parameters that play vital roles in affecting particle-cell interactions [9,10,11,12]. As for in vivo applications, these parameters also affect the traffic journey and bio-distribution of particles, especially when considering the complexity of the in vivo body environment. Based on the above-mentioned discoveries, one needs to bear in mind the importance of the developing micro/nano materials with better controlled features, which enables improved biomedical applications, especially for therapeutic delivery purposes.

In contrast to conventional synthesis methods, advanced manufacturing methods impose spatial and temporal control over the micro/nanoparticle fabrication process, introducing better controlled features, where the methods can be classified as top-down techniques in the micro/nanoparticle fabrication area (Figure 1B) [13,14,15,16,17]. The so-called advanced manufacturing technique employs computer or mechanical-aided systems and resources or uses automated material handling systems, robotics, and a computer controlled or an integrated manufacturing system [18,19]. Thus, this type of manufacturing technique can offer a great selection of material design, allowing for versatile control over the above-mentioned material features. Conventionally, advanced manufacturing techniques mainly focus on enhancing worker efficiency as well as worker control over mechanized designs [17,19]. In the past 10 to 20 years, advanced manufacturing techniques, including photolithography, e-beam lithography, soft lithography, layer-by-layer assembly, etc., were explored for generating micro/nano materials with controllable features [17]. Thus far, these materials have been widely studied in biomedical applications including drug delivery, cell surface engineering, cell tracking, vaccine development, and bio-imaging, showing dramatic advantages compared to conventional spherical micro/nano materials [10,20].

This paper focuses on reviewing the existing advanced manufacturing techniques of well-controlled micro/nanoparticles for therapeutic delivery purposes. The review starts by examining the major characteristics of micro/nano particles including size, shape, component materials and surface properties, followed by the existing advanced manufacturing techniques for producing controllable micro/nanoparticles. Considering the fast growth of the biomedical engineering field, we mainly focused on the therapeutic (i.e., drug and vaccine) applications of these materials.

## 2. Key Characteristics of Nanomaterials for Therapeutic and Imaging Applications

Micro/nano technology projects a promising future of benefiting our society through promoting the development of modern medicine. Current academia and industrial researchers believe that micro/nano technology could broadly impact the medical fields and potentially save numerous lives in future applications [21,22]. In human medicine, micro/nanotechnology is turning medicine from passive structures to active ones; researchers, physicians and medical teams accomplish this by using more “smart” strategies such as targeted delivery, controlled release, etc. [23,24,25]. Compared to conventional strategies, new therapies based on micro/nano technology illustrate fewer side effects and greater effectiveness [23,24,25]. Micro/nano technology is also aiding our understanding and inferring of the human biological system at the molecular level [26,27]. Despite these promising future applications, current commercially available micro/nano technology-based products for in vivo applications are very limited. This is mainly due to two reasons: first, the in vivo human biological system is very complicated, thus prohibiting our understanding the impact of micro/nano materials; second, the major characteristics of micro/nanoparticles, including size, shape, surface properties and component materials, would affect their interactions with biological entities such as cells, tissues and organs both at local and systemic levels [28,29]. Thus, in this section, we review the roles of micro/nano particle characteristics in the in vitro and in vivo biomedical applications, especially for therapeutic delivery purposes.

### 2.1. Size

The size of micro/nano materials directly impacts the in vitro and in vivo applications [28,30]. In vitro, size significantly influences the uptake efficiency and internalization mechanism of particles by cells. For example, in one study, researchers demonstrated that particles with a diameter of 100 nm had 2.5-fold greater uptake efficacy by Caco-2 cells than the 1 µm particles (10 times larger) and 6-fold higher uptake efficiency than 10 µm particles (100 times larger) [31]. Upon uptake, the nanoparticles will be trafficked to the endosome-lysosomal pathways, or the nanoparticles can be delivered to the lysosomes via vesicles, depending on the cellular types and the size of nanomaterials [32,33,34]. In non-phagocytic cells, particles at nanoscale (i.e., smaller than 1000 nm) can also be internalized by the cells, but the uptake of materials at the micron scale is difficult [35]. Beyond internalization efficiency, particle size also affects the internalization mechanisms by cells. For example, researchers reported that micro/nano materials with sizes larger than 500 nm are internalized by macrophages via phagocytosis, and particles smaller than this size can be internalized by pinocytosis [36]. Of note, no radical discontinuity exists between pinocytosis and phagocytosis, but phagocytosis tends to play a major role when particle sizes are larger than 500 nm [37]. Upon being internalized into cells, particle size could affect the internalization pathway and the subsequent cellular routing [35]. In one example, nanomaterials with diameters smaller than 200 nm were internalized by clathrin-coated pits; the ones with diameters around 500 nm were usually involved in caveolae-mediated internalization [35]. In another example, nanomaterials with diameters around 200 nm were easily delivered to the lysosomes within cells, but those with diameters around 500 nm were more difficult to be routed to lysosomes [35]. Beyond the in vitro impact, micro/nanoparticles also play vital roles in the in vivo applications. 

Following in vitro studies, it is equally important to characterize how the size affects the in vivo circulation, distribution and degradation of micro/nano particles. In one study, it was reported that nanomaterials with sizes larger than 200 nm were more easily trapped in the injection sites; nanomaterials smaller than 200 nm could be trafficked to the lymph nodes—a lymphatic organ that naive immune cells (i.e., T cells and B cells) reside in [35,38,39,40]. Upon trafficking from the injection site to the lymphatic system (vessels and lymph nodes), the vaccine materials activate these naive immune cells for promoting the activation of the body’s immunity [35,38,39,40]. With respect to in vivo bio-distribution, researchers found that nanomaterials smaller than 500 nm circulated throughout the body. For effective and expanded in vivo circulation, the size of rigid materials needs to be smaller than 10 μm—the diameter of the smallest capillaries [41]. People injected particles with a diameter of 4–5 μm into mice carotid arteries without producing any detectable problems; a majority of these particles with sizes ranging from 1 to 5 μm were eventually trapped in the liver tissue [42,43]. Based on the advantages of smaller-sized particles for in vivo circulation, researchers designed large particles for clinical settings, including the occlusion of blood vessels that connect tumor tissues. By blocking these tumor connecting vessels, the supply of oxygen and nutrients to tumor tissues would be greatly limited, prohibiting the progression of tumor growth [44]. For air-borne micro/nano materials targeting the pulmonary system, a diameter ranging from 0.5 to 5 µm would ensure a successful delivery deep into the lungs, but particles with a diameter smaller than 0.5 µm would be exhaled [45,46,47]. In addition to circulation and distribution, particle size influences the in vivo degradation of micro/nano materials. In brief, size influences particles’ in vivo hydrolysis by controlling the penetration depth of water into the materials. As a result, the hydrolysis-based factor could determine the bio-degradation of materials and the release of therapeutic cargos from nanomaterials. It is thus believed that the size is a key parameter impacting the therapeutic efficacy of the treatment [48].

### 2.2. Surface Property

Surface property is the second parameter that affects the interactions between nanomaterials and cells/tissues [49,50]. Briefly, this parameter influences the internalization efficiency of micro/nano materials by cells and the in vivo circulation. In one study, it was revealed that a primary amine group modified on a silica particle surface improved the effectiveness of internalization as compared to other functional groups (i.e., sulfate, hydroxyl, and carboxyl groups) [49]. This is primarily because cell surfaces are negatively charged, and primary amine groups are usually positively charged, which prevents repulsion between the two surfaces. Using this property, researchers utilized a positively charged polymer thin film with nano-scaled thickness to modify and engineer cell surfaces for cell based drug delivery [51].

In addition to in vitro applications, negatively charged surfaces can also reduce adsorption of plasma proteins (the majority of which carries negative charges) on micro/nano materials which would otherwise impede their in vivo circulation half-life. Meanwhile, negatively charged surfaces tend to diminish non-specific internalization of micro/nano materials by immune cells, thus enhancing their circulatory capacity [49]. For biomedical applications, surface property modifications of micro/nano materials can also benefit cancer treatment. Existing studies demonstrated that a positively charged surface can lead to an enhanced accumulation of micro/nano materials in tumor tissues compared to negatively charged ones. In addition, the positively charged surfaces could also lead to an increased retention time in tumor tissues [49,52]. A similar trend was demonstrated in the design of radio-sensitive nanomaterials, where a positively charged surface resulted in an enhanced accumulation of X-ray responsive materials in cancer cells [53]. Beyond using the charged surfaces, different surface properties were also employed to promote specific interactions between particles and cells. For example, the anti-DEC 205 receptor—a receptor that controls immune cell activation—was used to modify material surfaces to promote the secretion of certain cytokines by dendritic cells and T cells [54]. In the same study, researchers also reported that the density of the anti-DEC 205 receptor was associated with cytokine secretion levels; the denser the receptor concentration, the higher the promoted cytokine level [54]. Taken together, these studies demonstrate the importance of surface properties on circulation effectiveness and therapeutic efficacy of micro/nano materials.

As expressed previously, surface property can affect the in vitro and in vivo destination of nanomaterials. Based on this phenomenon, different types of chemistry have been employed to modulate nanomaterial surface properties to improve their biomedical applications. For example, polyethylene glycol (PEG) was widely used to reduce the non-specific in vitro and in vivo adsorption of proteins onto materials [55,56]. In one study, it was reported that PEG modified liposomes had enhanced in vivo circulating half-life compared to unmodified liposomes [57]. For cancer treatment, the PEG modified materials can accumulate in tumor tissues preferentially as compared to unmodified materials, mainly due to the enhanced permeability retention (EPR) effects [58,59]. In biomedical applications, for another instance, the grafting of certain functional groups on the surface can promote the interactions between nanomaterials and specific cell or tissue. To name a few, examples of these chemicals include antibodies, carbohydrates, and peptides for targeting cells like intestinal M cells and tissues including brain, liver, and tumors [44]. Overall, the relevant studies conclude that the control over micro/nano materials surface properties is vital to improve the implementation of biomedical applications.

### 2.3. Component Materials

Component materials of micro/nanoparticles is critical for directly determining the feasibility of in vitro and in vivo biomedical applications. Materials intended for biomedical applications, such as drug delivery and vaccine development, need to be minimally toxic, biocompatible, and biodegradable. Upon in vitro and in vivo applications, the component materials affect their degradation mechanism/rate, and the release of therapeutic cargos from the materials [41]. A broad range of materials have been employed for drug delivery and vaccine development including synthetic polymers, stimuli responsive materials, and biological materials-components that are extracted from biological units such as cells and tissues. Synthetic polymers are featured by their outstanding flexibility in controlling their composition, property, and structure. Representative polymeric materials for constructing micro/nano materials for drug/vaccine development include poly(lactic acid) (PLA), poly(glycolic acid) (PGA), and their co-polymer PLGA. Of note, PLGA has been approved by U.S. Food and Drug Administration (FDA) for in vivo use. In these applications, degradation of PLGA nanomaterials could be tailored by changing the molar ratio of lactic acid and glycolic acid [60]. In addition to PLGA, researchers have used other synthetic polymers such as polyorthoesters, polyanhydrides, polyamides, polyphospha-zenes, and polyphosphoesters to develop nanomaterials for therapeutic cargo deliveries [61]. Importantly, these materials allow prolonged and controlled release of therapeutic cargos, targeting, and co-delivery of different therapeutic components for specific cells/tissues.

“Smart” materials—materials that are responsive to certain environmental or chemical stimuli (i.e., temperature, pH, or specific chemicals)—were also explored to produce nanomaterials for different biomedical applications [62,63]. For example, poly(N-isopropylacrylamide) (PNIPAM) is hydrophilic at room temperature and hydrophobic when the temperature is above a lower critical solution temperature (LCST). By utilizing this unique feature, researchers demonstrated the potential biomedical applications including drug/enzyme release and biosensors [64]. In addition to PNIPAM, other smart materials including pH-sensitive materials such as poly(L-histidine)-PEG and poly(L-lactide)/PEG-polysulfonamide (PLLA/PEG-PSD) have been applied for drug delivery applications. Of note is that, upon intracellular uptake, the pH-sensitive materials can disrupt lysosomes in low pH environment, thus protecting and minimizing the degradation of cargo agents in lysosomes [62]. In vaccine development, pH-sensitive materials can promote the delivery of antigen materials to the cytoplasm of antigen-presenting cells (i.e., dendritic cells). The delivered antigen will then be presented in the histocompatibility complex (MHC) class I molecules on the antigen presenting cells. This is a vital step for the activation of cytotoxic T cells for killing the invaded bacteria, virus, and even certain cancers [23]. By utilizing “smart” materials, biomedical diagnostics and therapies can be developed for efficient and targeted therapeutic delivery.

Compared to synthetic materials, biological materials offer unique advantages in developing micro/nanoparticles for biomedical applications according to high degree of biocompatibility and biodegradability [41]. Representative examples of biological materials include polysaccharides, DNAs, proteins or peptides, liposomes, etc. Polysaccharides are highly versatile in composition, structure, and property, and thus are widely employed for drug delivery [57,58]. Using this group of materials for biomaterial vaccine development, studies reported that polysaccharides have adjuvant effects—a function to amplify the immune functions of the vaccines [65,66]. DNA contains genetic information and is usually employed as a therapeutic component for gene therapy. Since DNA is negatively charged in nature, researchers used positively charged materials including chitosan and poly(ethylene imine) (PEI) to interact with DNA to form nanoparticles for gene delivery [67,68]. Additionally, peptides such as IFN-γ, IL-6 are now being explored to regulate specific immune responses for immune therapy, where these cytokine can produce innate and adaptive immunity against external invading infectious agents, and have been used to inhibit tumor growth [69]. In addition, lipids are another group of biomaterials for drug delivery and vaccine development via forming liposome-nanoparticles [70]. These cutting-edge biological strategies offered unique advantages for next-generation medicine and regulatory approval. In addition to the above-mentioned materials, metal nanoparticles such as gold and iron oxide nanoparticles are another group of materials that have been explored for drug delivery [6,58,71,72]. In one example, adjuvant and antigen materials were assembled onto gold nanoparticles to generate nano-vaccines that can induce controllable immunity against melanoma cancer [73,74]. In another, magnetic iron oxide nanoparticles were studied for drug delivery, magnetic resonance imaging (MRI), and tumor hyperthermia therapy [75].

Beyond metal nanomaterials, carbon-based materials, including carbon nanotubes, nano-diamonds, and C60 fullerenes are another type of carrier for drug delivery according to their unique dimensions and physiochemical characteristics. However, for carbon-based technology, biosafety remains a huge concern especially when these materials are employed in vivo [76,77]. In sum, by simply altering and/or combining unique component materials, researchers are paving the way to discover and optimize biomedical applications at the micro/nano-scale.

Beyond all the above-mentioned component materials, some micro/nano particle materials affect their in vitro and in vivo applications from surfaces. Examples include the materials that were mentioned in Section 2.3, including PEG, antibodies, aptamers, and dextran. Since these materials were already discussed, we did not include them in this section.

### 2.4. Shape

In recent years, the impact of micro/nano materials’ shape on their in vitro and in vivo applications have been increasingly recognized for both in vitro and in vivo applications [78]. In conventional bottom-up techniques, particle shape is usually spherical. This is determined by the spontaneous minimization of surface energy in the process of nanoparticle formation. Although spherical nanomaterials are desirable for drug delivery applications due to the minimized surface-to-volume ratio, the progress of micro/nano medicine requires generating non-spherical particles for a more robust therapeutic efficacy.

The development of advanced manufacturing techniques in biomedical applications has promoted the feasibility of generating non-spherical particles with well-controlled shapes. Compared to spherical materials, those with non-spherical shapes offer unique advantages in controlling the interaction between materials and biological units (i.e., cells, tissues, etc.) because the shape could affect the way particles attach to cells/tissues or their internalization by the cells. For example, disk-shaped particles have a larger binding area compared to spherical materials, leading to stronger force interactions between the materials and cells [79]. Particularly in vaccine development, shape would influence the way macrophages internalize materials [80]. In one study conducted by Mitragotri et al., they reported that the materials aspect ratio determines whether macrophages will phagocytose or spread on the particles [20,81,82,83]. In another study by Doshi et al., they demonstrated that spherical polystyrene materials can be phagocytosed by macrophages; in contrast, disk-shaped materials resisted phagocytosis [84]. With respect to intracellular delivery pathway, the DeSimone group reported that nanomaterials with different shapes had distinct intercellular delivery pathway [85]. The above studies provide evidence of how shape offers unique advantages for in vivo internalization and specification.

Shape could also affect the trafficking of materials in blood vessels, airways and gastrointestinal tract [81]. In most cases, the transport pathway of spherical materials is predictable because of the symmetrical geometry. In contrast, the traffic journey of non-spherical materials is more difficult to predict when they pass through filtering organs such as liver and spleen. An example is the disk-shaped red blood cell, which usually has a diameter around 7 µm and can routinely pass through the spleen. In contrast, synthetic spherical materials need to have a diameter smaller than 200 nm for filtering through the same organ [86]. Additionally, shape also affects margination. Briefly, disk-shaped materials have an analogous behavior compared to red blood cells when trafficking in blood vessels [87]. Rod-like materials experience lower drag forces and higher surface contact area as compared to a spherical material of similar volume [88]. It is worth mentioning that disk-shaped materials can easily pass through capillaries when compared with other shaped materials with similar dimensions, and have longer vasculature circulation half-life as compared to spherical ones [89]. Similar results were demonstrated by DeSimome et al.; this group showed a 30-fold increase in the elimination half-life for materials with shape mimicking blood cells [86]. These features make the materials with specific shapes competitive candidates for drug delivery and vaccine development.

### 2.5. Nanomaterial-Induced Cellular Toxicity

The side effects of micro/nanoparticles such as toxicity are a big concern, especially when these micro/nanoparticles are employed in vivo, where the materials need to interact with cells, tissues and organs. Existing studies have discussed this issue broadly, where the exposure of humans to micro/nanoparticles may induce some undesirable side effects [90]. In many cases, cellular toxicity caused by nanomaterials is caused the production of reactive oxygen species, which yield oxidative stress and inflammation response. The way these nanomaterials interact with cells also determines whether cells undergo autophagic, apoptotic, or necrotic cell death [90]. The properties of nano/microparticle discussed above also determine how cells respond to them and the consequent cellular toxicity. As mentioned above, size and shape can affect particle uptake efficiency and thus determine the amount of nanomaterials entering the cells. Existing studies reported that nanoscale size was a detrimental factor affecting the autophagic response; as a contrast, the aggregated nanoparticles show less tendency to induce autophagy [91,92]. Beyond micro/nanoparticle sizes, surface chemistry matters in the toxicity issues. For example, in one study, amine and carboxylic acid modified polystyrene nanoparticles can induce apoptosis in the Caco-2 cell monolayer while carboxylic acid-modified particles decreased cell viability faster and in a stronger manner [93]. In another study, cellular toxicity was correlated with the number of surface amine groups on polyamidoamine dendrimers (PAMAM) [94]. Li et al. reported that cationic PAMAM tends to induce autophagic cell death in the A549 cell line more than anionic ones [95]. In addition to these impacts, the toxicity of nanoparticles is also related to the cell types. For example, multiwall carbon nanotubes (MWCNT) can induce apoptosis in human skin fibroblast and T lymphocytes; in contrast, MWCNT induces necrosis in mouse macrophages [96,97]. In short, on the one hand, the cellular toxicity of micro/nanoparticles depends strongly on the cell types; on the other hand, we also noticed that the underlying toxicity mechanisms of micro/nanoparticles vary in the literature. Thus, this topic requires more in-depth studies to further illustrate the exact mechanisms.

## 3. Advanced Manufacturing Techniques in Designing Micro/Nano Particles for Therapeutic Delivery

As mentioned, through using computer or mechanical-aided systems and resources, or automated material handling systems, advanced manufacturing techniques impose spatial and temporal control to produce micro/nanoparticles with well-controlled features (i.e., size, shape, surface property and component materials). In the micro/nano particle fabrication field, these advanced manufacturing guided micro/nanoparticle fabrication methods were viewed as “top-down” techniques, which are named as a contrast to the conventional “bottom-up” methods. For therapeutic delivery applications, these well-defined micro/nano particles have dramatic advantages. In the following parts, we will introduce the major particle fabrication methods that involve the use of different types of advanced manufacturing techniques and discuss the therapeutic delivery applications associated with the micro/nanoparticles.

### 3.1. Photolithography

**Figure 2:**
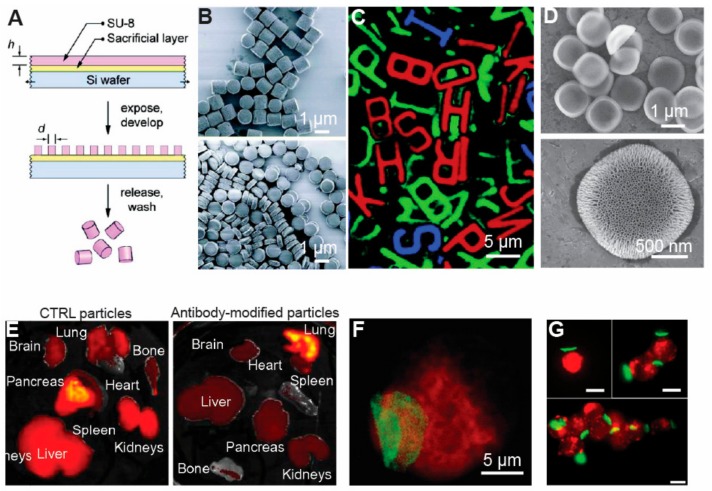
Photolithography-based techniques were employed to generate micro/nanoparticles with controllable features for delivering therapeutic cargos. (**A**) In photolithography-based particle production techniques, a layer of photoresist was coated onto a sacrificial layer (i.e., SU-8). UV light passed through a photomask with certain features onto the photoresist layer, with micro/nanostructures of the thin film defined by photomask features. Through dissolving the sacrificial layer, free micro/nanoparticles were produced [101], with permission from © American Chemical Society; (**B**) Photolithography-based techniques allowed the production of micro/nanoparticles with variable aspect ratios. Particles with two different aspect ratios were produced in this study [101], with permission from © American Chemical Society; (**C**) The versatility of photolithography-based particle production techniques was exemplified by producing particles of 26 Latin alphabet letters [103], with permission from © American Chemical Society; (**D**) Photolithography techniques were used to produce silicon microparticles with porous structures [105], with copyright permission from © Springer Nature; (**E**) The porous silicon in Figure 2D was used to treat mice in vivo. The unmodified particles accumulated in different organs such as the liver and pancreas, etc., whereas the antibody-modified particles mainly accumulated in the lung [107], with permission from © Elsevier; (**F**) Photolithography-produced microparticles with a disk shape were attached onto a monocyte cell surface. Such a platform will allow us to develop a cell-based delivery system by loading therapeutic cargo onto particles [111], with copyright permission from © Elsevier; (**G**) The photolithography technique was used to produce particles with a thin disk shape for assembling different number of cells. The cellular aggregates with a controlled number of cells could be employed for cell therapy. Scale bars were 20 µm if not specified [111], with permission from © American Chemical Society.

Photolithography relies on exposing UV light through a photomask with certain features onto a thin film of light-sensitive materials, typically generating micro/nanostructures that are defined by the features of the photomasks (Figure 2A) [98,99,100,101]. Materials that have been used in photolithography for generating micro/nano structures include photoresists, silicon, poly(methyl methacrylate) (PMMA), hydrogels, etc. [102]. Using these materials, studies demonstrated the feasibility of generating micro/nanoparticles with variable aspect ratios and shapes (Figure 2B) [101]. Photolithography also enables generating particles with versatile 2-D shapes, as exemplified by the microstructures with the shapes of the 26 Latin alphabet letters (Figure 2C) [103]. Through using a double photolithography process and other manufacturing techniques (i.e., plasma treatment), photolithography techniques allow the production of micro/nanoparticles with complicated 3D structures [103].

Micro/nanoparticles generated via photolithography techniques have been widely employed in biomedical applications, especially for delivering therapeutic cargos. In one study, the Ferrari group produced disk-shaped silicon microparticles with mesoporous structures (Figure 2D) [104]. The mesoporous structures allowed loading of SiRNA, MRI contrast agents, and thermal responsive materials into the pores, which enhances the loading capacity of the particles as compared to solid particles with no pores (Figure 2D, lower figure) [105,106,107]. In vivo, the Ferrari group tested the biosafety of these mesoporous silicon micro/nano particles via intravenous injection. Briefly, their study illustrated the mesoporous silicon micro/nano particles did not change the levels of renal and hepatic biomarkers in plasma, neither did they affect the level of 23 plasma cytokines [108]. Additionally, the mesoporous micro/nano particles did not change the hepatic markers in the liver and spleen or result in filtration of leukocytes into major organs including the liver, spleen, kidney, lung, brain, heart and thyroid [108]. In a mice pancreas cancer treatment study, antibody-modified silicon mesoporous particles were employed to target tumor tissue. Compared to unmodified particles, antibody-modified silicon mesoporous particles were able to target pancreas tissues more effectively (Figure 2E) [108]. Additionally, compared to conventional spherical particles, the microdisks had improved in vivo circulation and targeting due to the unique shape [108]. These studies thus demonstrated particle shape can be harnessed to aid delivery of therapeutic cargo with minimal disruption of major organ functions.

In addition to porous silicon particles, photolithography techniques were used to generate disk-shaped micro/nano particles composed of polyelectrolyte materials for drug delivery and cell function control. In one study, the Rubner group attached these micro/nano particles onto single mammalian cell surfaces, or bound a certain number of cells into cellular aggregates (Figure 2F,G) [109]. In an early study, this group loaded magnetic nanoparticles into the microstructures, which could precisely control cell trafficking pathways under a magnetic field [109]. By loading anticancer drugs such as doxorubicin (DOX) into the disk-shaped microparticles, the particles have been used for cancer treatment in culture [110]. Compared to conventional spherical particles, one advantage of these disk-shaped microparticles is that they can resist phagocytosis from macrophages. Using this interesting property, the Rubner and Mitragotri groups attached these disk-shaped particles onto monocyte surfaces, where the circulation of these surface-modified monocytes to inflammatory tissues in mice controlled inflammation [111]. It is worth emphasizing that the surface-modified monocytes penetrated target tissues (i.e., skins and lungs), without losing their functions during circulation (Figure 2F,G) [111]. From a therapeutic delivery perspective, these studies illustrate that advanced manufacturing techniques allow generating well-controlled micro/nano particles to engineer cell surfaces for cell-based drug delivery [112]. These studies also demonstrated the feasibility of using cell-mediated therapies to broadly target diseased tissues. Additionally, for drug delivery purposes, multiple types of therapeutic cargos may be loaded into the disk-shaped particles. Upon circulating inside the body, the cells can deliver the therapeutic cargos to specific targeting tissue sites, creating a versatile platform for cell-based drug delivery. Compared to using rigid spherical micro/nanoparticles for targeted delivery, these cell-based delivery systems can more easily penetrate different tissues and organs (e.g., capillaries, skins, lungs, etc.) This is because for successful in vivo circulation, the rigid micro/nanoparticles need to be smaller than the diameter of capillaries (i.e., 10 µm in diameter). Beyond these potential advantages, most biomaterials generate certain levels of immune responses, including changed cytokine secretion upon the in vivo administration of materials [113,114]. Using these advanced manufacturing techniques-enabled cell delivery systems, the undesirable immune responses can be minimized. However, despite all the above-mentioned advantages of photolithography, a broad use of this technique in the biomedical field is usually limited by the requirement of extremely expensive clean-room based equipment. To solve this cost issue, other advanced manufacturing techniques such as soft lithography can assist us to generate micro/nano particles with precisely defined features at a lower, more reasonable cost.

### 3.2. Soft Lithography

Soft lithography is another advanced manufacturing technique that has been explored for producing micro/nano particles with controllable features [115,116,117,118]. Typically, the soft lithography technique employs a soft polymer stamp to print a monolayer of materials (i.e., polyelectrolytes, DNAs, proteins, nanoparticles, etc.) onto different surfaces (i.e., glass, silicon substrates, etc.) [119]. Beyond generating a monolayer of materials, soft lithography techniques have also been employed to generate micro/nano particles with controllable features. For example, in one study, Guan et al. used polymer stamps with micro-wells or micro-pillars on the surfaces to produce polymeric particles with controllable features [120,121]. Briefly, in this work, polymer materials such as PLGA and poly(methyl methacrylate) (PMMA) were brushed onto polydimethylsiloxane (PDMS) polymer stamp surfaces, followed by printing the stamp onto a flat surface coated with a thin layer of polyvinyl alcohol (PVA). Materials in the micro-well or on the micro-pillar of the stamp were then printed onto the flat PVA surface as a material array. Once the PVA layer was dissolved with water, the materials were released as free disk-shaped particles. In a similar study, instead of using a PDMS polymer stamp, researchers employed a hydrogel template as a mold to generate micro/nanoparticles ranging from 200 nm to 10 µm [122]. Compared to a polymer mold, the hydrogel mold is overall mechanically weak. However, hydrogel materials allowed for versatile sol-gel phase transition upon changes in environmental conditions; thus it enabled the release of the micro/nanoparticles in a sustained and environmentally friendly way [123,124].

**Figure 3:**
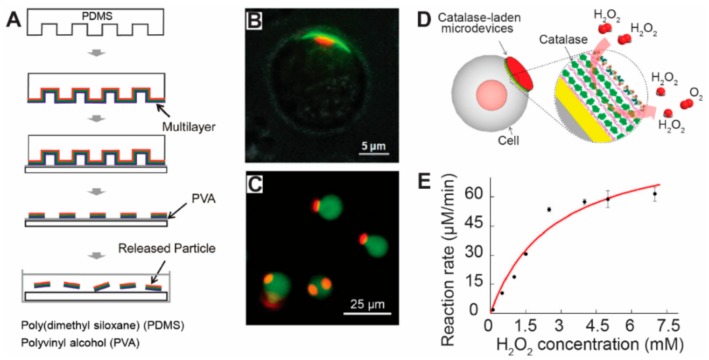
Soft lithography techniques were employed to generate micro/nanoparticles with controllable features for therapeutic delivery to cells. (**A**) Soft lithography techniques were used to generate disk-shaped micro/nanoparticles. Polyelectrolyte multilayers were assembled onto a soft PDMS polymer stamp. The stamps were then printed onto a substrate coated with PVA film, transferring the multilayer on the protruding pillars of the stamp onto the PVA film. Multilayer disk-shaped particles were produced by dissolving the PVA film with water [51], with copyright permission from © John Wiley and Sons; (**B**) Dot-on-pad micro/nanoparticles were attached onto a mammalian cell. The red dots were thermoplastic materials with dye. The green materials were the pad loaded with dye [125], with copyright permission from © John Wiley and Sons; (**C**) Disk-shaped particles were attached onto the cell surface. The red color indicates the particles with dye, and the green color represents the cells stained with another dye [128], with copyright permission from © American Chemical Society; (**D**) A schematic picture showing a cell-based drug delivery system. Catalase were laden into the disk-shaped particles. The catalase will react and transform H_2_O_2_ into O_2_ [128], with copyright permission from © American Chemical Society; (**E**) The reaction rate of the catalase depends on the concentration of H_2_O_2_ surrounding the cells in culture medium [129], with copyright permission from © American Chemical Society.

In addition to using soft or solid molds, soft lithography-based techniques allow integration of other manufacturing techniques to generate micro/nano particles with controllable features. In one example, Zhang et al. produced micro/nanoparticles by integrating soft lithography with layer-by-layer assembly—a technique that assembles positively charged and negatively charged materials via electrostatic interactions into a nano film [51]. Figure 3A illustrated the procedures of this approach. Briefly, on a soft-polymer stamp with certain topological features, multilayers of materials are assembled into a nano-film via electrostatic interactions. These micro/nano particles were around 500 nm to 10 µm in diameter and nanometer scale in thickness [51]. For drug delivery applications, the micro/nano particles fabricated by this technique were attached onto cell surfaces via electrostatic interactions (Figure 3A) [51]. A 72 h microscopic observation illustrated that the microparticles attached on the cell surfaces did not change cellular viability [51]. In another study, Zhang et al. used a similar approach to produce micro/nano particles with a dot-on-pad structure, as illustrated in Figure 3B [125]. In these particles, the red dots were composed of thermoplastic materials (i.e., PLGA) containing fluorescence dye, and the green pads were made of polyelectrolyte multilayers containing another fluorescence dye (Figure 3B). By controlling the surface property of the polyelectrolyte multilayers, the dot-on-pad particles can attach to the cell surface in a unidirectional manner, allowing the therapeutic agents loaded in the dots to be released to the cells in a controlled direction (Figure 3B) [125,126]. Continuing on this work, this research group loaded gold nanoparticles into the multilayer microparticles. By labeling the gold nanoparticles with Raman reporters, the multilayer micro/nanoparticles were employed for Raman labeling of cells [127]. Compared to using single gold nanoparticles for Raman labeling, the multilayer particles created by Zhang et al., highly enhanced the Raman signal due to the loading of millions of gold nanoparticles within the multilayer structure [127]. These studies demonstrated that advanced manufacturing techniques can offer more versatility and flexibility to improve current biomedical therapies and applications.

For drug delivery applications, microparticles fabricated by soft-lithography and other manufacturing techniques were loaded with therapeutic agents such as catalase and were attached to cell surfaces for a cell-based delivery system (Figure 3C) [128]. In this design, researchers attached the catalase laden disk-shaped particles onto mammalian cell surfaces via electrostatic interactions (Figure 3C). During cell culture or in vivo circulation, the catalase within the particles interacted with H_2_O_2_ (hydrogen peroxide, i.e., an oxidizer that is toxic to cells), generating O_2_ that can improve cell viability and whole body health (Figure 3D) [128]. It is worth mentioning that the multilayer structure in the particles allowed one to precisely control the amount of catalase loaded into the particle. Importantly, this study found that the classic Michaelis-Menten model fit well with the reaction rate data, thus providing the tool to predict enzymatic degradation rate of H_2_O_2_ at any given concentration. (Figure 3E). This study also demonstrated that the particle retained enzyme activity after a one-weeks incubation period [128]. Together, the above studies illustrated the advantages of not just one advanced manufacturing technique but a combination of approaches with soft-lithography, which allow for a higher impact and broader utilization in the therapeutic delivery field.

### 3.3. Nano-Imprint Lithography

**Figure 4:**
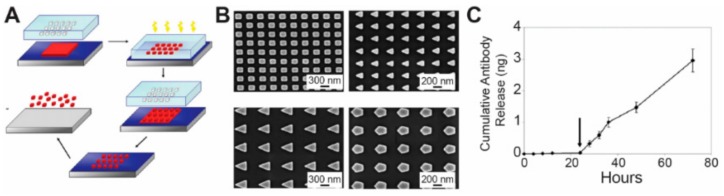
Nanoimprint lithography (NIL) techniques were used to generate micro/nanoparticles with controllable features for therapeutic delivery to cells. (**A**) NIL relies on printing a mold onto a layer of photo or thermal materials. UV light was used to transform the materials into a solid shape. The residual materials during printing will connect the nanoparticles together and thus require reactive ion etching techniques to remove the residual layer, generating isolated nanoparticles [131]; (**B**) NIL based techniques were able to generate nanoparticles with different shapes. Four shapes were illustrated in this work [130]; (**C**) The NIL-based nanoparticle production techniques allow the cumulative release of therapeutic cargos. These techniques also allow the loading of smart materials (i.e., enzyme triggered materials), thus the cargos can be released in a certain enzyme environment [130], with copyright permission from © Elsevier.

Nanoimprint lithography (NIL) is a high-resolution technique that allows for generation of wafer-scale nano-patterns on substrates at a low cost [129]. In this technique, a layer of thermal or light-responsive material is coated onto a substrate, where a rigid mold was used to replicate its topological features onto the material layer. For thermal-based materials, NIL started with printing the mold against a layer of thermoplastic materials coated on the substrates, with the temperature controlled above the glass point of the materials (i.e., for a liquid or soft state). A cooling process was then employed to turn the thermoplastic materials into a solid state to replicate the topological features of the mold onto the materials [130]. In the light-based NIL, a transparent mold was pressed against a film of light-responsive materials coated on a flat substrate, followed by using light at certain wavelength, e.g., UV light, to convert the light-responsive materials into certain features. (Figure 4A) [130].

Existing nanoimprint technology allows the production of structures with a resolution down to 2 nm. In addition to its high resolution, NIL is also featured by its high throughput at a low cost; thus industry views NIL as a very promising technology in the semiconductor field [129]. In NIL, all the detailed topological features of the imprint mold or mask, including the defects and surface roughness, will be replicated, making the quality of the initial imprint mold a vital issue for the production. These masters or molds in NIL are usually created by electron beam lithography or photolithography, which are typically expensive when a large mold surface area is required [131,132]. The cost of NIL can be reduced via other technologies such as interference lithography, although the price is still relatively high; interference lithography can produce features around 50 nm at wafer-scale which is an applicable dimension for biomedical applications since nanomaterials at this size could efficiently circulate in vivo [71,72].

For biomedical applications, researchers utilized NIL to produce monodisperse nanoparticles with precise sizes and versatile shapes (Figure 4A) [130]. In one example, a nanoimprint mold was employed to press against a thin layer of UV-responsive polymer materials coated on silicon substrate. Upon UV exposure, the polymer materials were reacted to form solid nano structures. To generate monodisperse nanoparticles, oxygen plasma was employed to remove the residual layers (Figure 4A). This technique allowed generating nanoparticles with well-defined shapes (i.e., circular, triangular, rectangular, etc.). Sizes of the nanoparticles produced by this technology were precisely controlled as well, ranging from 50 to 200 nm (Figure 4B). For therapeutic delivery purposes, the nanoparticles in this work were laden with antibodies and nucleic acids. It is worth mentioning that these nanoparticles were designed to be “smart” by adding enzymatically triggered materials into the particles. Upon enzyme-triggering, antibodies and nucleic acids loaded within the nanoparticles were released in a controlled accumulative manner, offering a potential approach for a smart-controlled release of therapeutic cargos such anti-cancer drugs and vaccine components (Figure 4C). This work illustrated the feasibility of using NIL to design nanoparticles with controllable features for drug and gene delivery. Beyond this work, multiple studies employed NIL for designing nanostructures with controllable features [129,133]. However, NIL involves using a heating or UV exposure step to solidify the materials into micro/nano structures. Additionally, NIL usually requires a reactive ion etching step to remove the material residues left by the printing process, which usually connects the nano-features together instead of generating isolated nanoparticles. These UV exposure and the reactive ion etching steps could potentially damage the therapeutic cargos such as DNA and peptides loaded in the nanoparticles, thus limiting the application of this technology for therapeutic delivery purposes. To solve these challenges, the DeSimone group developed another technique named Particle Replication In Non-wetting Templates (PRINT) [85].

**Figure 5:**
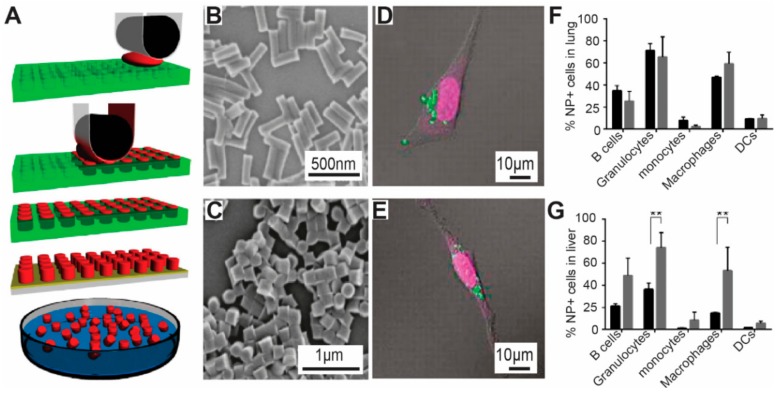
PRINT techniques were used to develop nanoparticles for delivering therapeutic cargos. (**A**) In PRINT technology, photo-curable perfluoropolyether (PFPE) molds were employed to emboss the materials for producing particles. The PFPE materials have a low surface energy and thus allow the materials to stay in the nano-cavities specifically [85]; (**B**,**C**) PRINT techniques allow generation og nanoparticles with controllable shapes and variable aspect ratios. The particles in (**B**) have a high aspect ratio and those in (**C**) have a lower one [85]; (**D**) Hela cells can readily internalize 3 µm cubic PRINT particles; (**E**) Similarly, Hela cells also take up cylindrical particles (3 µm in height). Comparing the internalization of cubic and cylindrical particles, Hela cells internalize cubic particles more effectively than cylindrical particles [85], with copyright permission from © (2008) National Academy of Sciences; (**F**,**G**) Using PRING particles, the DeSimone group illustrated that tumor tissues changed the level of different immune cells in mice. Briefly, tumor-bearing mice (gray bar) have a higher level of macrophages in lung compared to naive mice (black bar) [136]; (**F**) In the liver tissue, tumor bearing mice have a higher level of B cells, granulocytes, monocytes and macrophages than naive mice (black bar) [136], with copyright permission from © American Chemical Society. With the PRINT particles with controllable features, this work for the first time illustrated that change of body immunity by tumor environment.

PRINT is a manufacturing technique that allows the production of monodisperse micro/nano particles with controllable size, shape, and modulus. As illustrated in Figure 4A, in PRINT technology, photo-curable perfluoropolyether (PFPE) molds were used to emboss the materials for generating particles. PFPE materials have a low surface energy, thus allowing filling the micro/nano cavities in the mold selectively, without inducing the wetting phenomena in the micro/nano cavities. This non-dewetting property of PFPE thus allows the generation of isolated micro/nano particles without forming the interconnecting flash layer as occurs in the conventional imprint lithography (Figure 5A) [85]. Using this technique, a broad range of materials has been employed to generate micro/nano particles with precisely controlled shapes, sizes and aspect ratios, including poly(ethylene glycol), poly(d-lactic acid), poly-(pyrrole), and a triacrylate resin, hydrogels and biomaterials such as proteins (Figure 5B). With the micro/nano particles fabricated by PRINT technology, the DeSimone group explored the impact of nanoparticle shape on cellular internalization [85]. The study identified that Hela cells can readily internalize cubic and cylindrical micro/nano particles, with dimensions up to 3 μm. Importantly, the study showed that the cylindrical particles had a higher internalization efficiency compared to the cubic particles of similar size. Specifically, the study revealed that the internalization kinetics of the cylindrical nanoparticles by HeLa cells depended on the aspect ratio of the particles, where high aspect ratio particles (i.e., aspect ratio = 3) had almost a 4 times higher internalization rate than low aspect ratio ones (i.e., aspect ratio = 1) (Figure 5D,E) [85]. This kind of information is notably significant for designing the next generation of nanomedicine since it allows us to optimize the delivery and internalization efficiency of the therapeutic cargos. Beyond the fundamental internalization study, nanoparticles developed by the PRINT technology have been employed for other therapeutic purposes, including drug delivery and vaccine study [134,135,136]. For example, in one study, the DeSimone group used PRINT nanoparticles to investigate the impact of a tumor on local and global immune systems in vivo, where the study showed that the tumor environment can upregulate the clearance rate of nanoparticles [135]. Additionally, this study illustrated that the presence of tumors increased the amount of M2-like macrophages, which cleared the nanoparticles, leading to an increased accumulation of nanoparticles in organs such as liver and spleen (Figure 5F,G) [135]. This study thus highlighted the importance of studying the relation between immune status and nanoparticles, indicating a requirement of understanding immune function change for efficient drug delivery.

### 3.4. Mechanical Stretching

**Figure 6:**
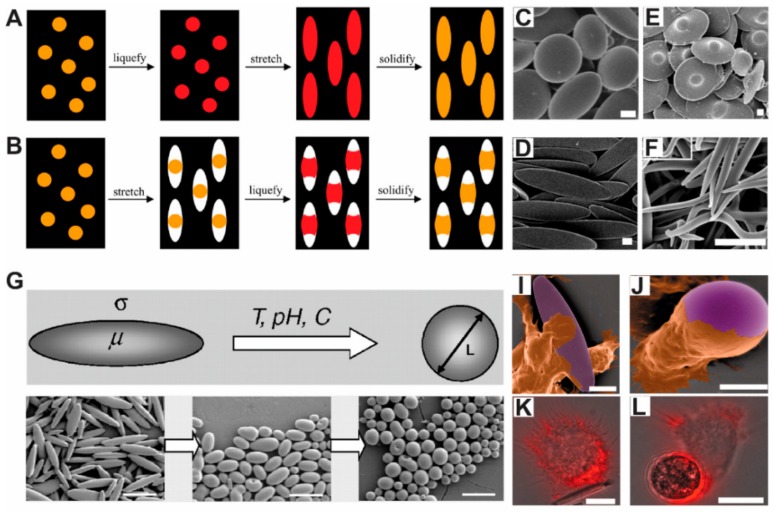
Mechanical stretching techniques were used to produce micro/nanoparticles for delivering therapeutic cargos. This group of techniques can be classified into two methods: (**A**) In the first method, thermoplastic spherical particles (i.e., polystyrene or PLGA) were embedded in a thin film of PVA. The particles were liquefied by heat or organic solvent, followed by stretching the PVA film with mechanical forces to create extra space, where the liquefied materials will fill in the space. Last, a cooling step was used to solidify the particles [83]. Copyright (2007) National Academy of Sciences; (**B**) In the second method, the PVA film embedded with thermoplastic particles was first stretched to generate some extra space for the particles, followed by liquefying the particles with organic solvent or heat to fill into the space. Particles were solidified by evaporating the solvents or lowering temperature [83], with copyright permission from © (2007) National Academy of Sciences. These techniques can generate particles with variable shapes such as (**C**) disk shape; (**D**) disk shape with a dot in the center; (**E**) elliptical shape; and (**F**) needle shape [83]. with copyright permission from © (2007) National Academy of Sciences; (**G**) Researchers studied the major factors that affect particle shapes including heating temperature, pH and particle viscosity and cooling speed [82], with copyright permission from © (2007) National Academy of Sciences. Using these particles, the researchers illustrated the impact of shape on the internalization of particles by macrophages. Artificially color-enhanced mages of macrophages internalizing (**I**) elliptical and (**J**) spherical shape particles. Fluorescence microscope showing the internalization of (**K**) elliptical and (**L**) spherical particles by macrophages [20], with copyright permission from © (2006) National Academy of Sciences. This study illustrated that the contact point between particles and macrophages will affect the internalization speed, where macrophages take up spherical particles more easily than elliptical particles of similar diameter.

Mechanical stretching is another manufacturing technique for producing micro/nano particles with controllable features. Briefly, the mechanical stretching techniques for making particles with different shapes can be divided into two schemes (Figure 6A,B) [83]. In the first method, thermoplastic spherical particles were suspended in PVA solution. The particles in solution were then coated onto a substrate, with the spherical nanoparticles embedded in the PVA film. The thermoplastic spherical particles were then changed into a liquid form either by using an organic solvent or heat (i.e., raising temperature above the glass transition point of the thermoplastic materials), followed by solidifying the thermoplastic materials by evaporating the organic solvent or reducing the temperature (Figure 6A) [83]. 

In the second method, the PVA film was first stretched by mechanical forces, creating a void space around the particles. The particles were then liquefied by using organic solvent or heat, filling the whole void space. By evaporating the organic solvent or lowing the temperature, the thermoplastic liquid was solidified into particles with sizes and shapes defined by cavities (Figure 5B) [83]. In these techniques, shapes of the particles were controlled by several parameters such as particle viscosity, PVA film thickness etc. Materials that have been employed in this technique include polystyrene, PLGA, with 20 different particle shapes created (Figure 6C–F). In a later study, the authors investigated the major forces behind the above particle shape changes, identifying that the final shape of the nanoparticles was controlled by several factors including the polymer viscosity and interfacial tension of the materials (Figure 6G) [82]. Using the clearly revealed mechanism, this technology produced elliptical disk-shaped particles and discovered disk-shaped particles with a diameter of 6 µm or other nanomaterials with high aspect ratio [82,137]. With the microparticles generated from this technique, this group demonstrated that 6 µm disk-shaped particles can resist phagocytosis by macrophages. As a contrast, conventional spherical-shaped particles with diameter smaller than 6 µm (i.e., low aspect ratio) were quickly phagocytosed by macrophages (Figure 6H) [20]. This study thus illustrated that micro/nano particles produced via the mechanical stretching technique can be explored to manipulate the interactions between materials and cells. Using the same technique, Meyer et al. designed surface modified ellipsoidal nanoparticles for developing artificial presenting cells. Compared to conventional spherical nanoparticles, ellipsoidal nanoparticles can reduce the internalization by macrophages [138]. In vivo, the ellipsoidal nanoparticles had a longer half-life compared to the spherical nanoparticles of the same diameter [138]. Beyond these advantages, the ellipsoidal nanoparticles stimulated antigen specific CD8^+^ T cells—immune cells that can be harvested to combat against cancer or invaded bacterial [138]. Thus, this work, together with others, presented us unique examples of using advanced manufacturing techniques for generating non-spherical particles for drug delivery and vaccine development applications.

### 3.5. Microfluidic Fabrication

**Figure 7:**
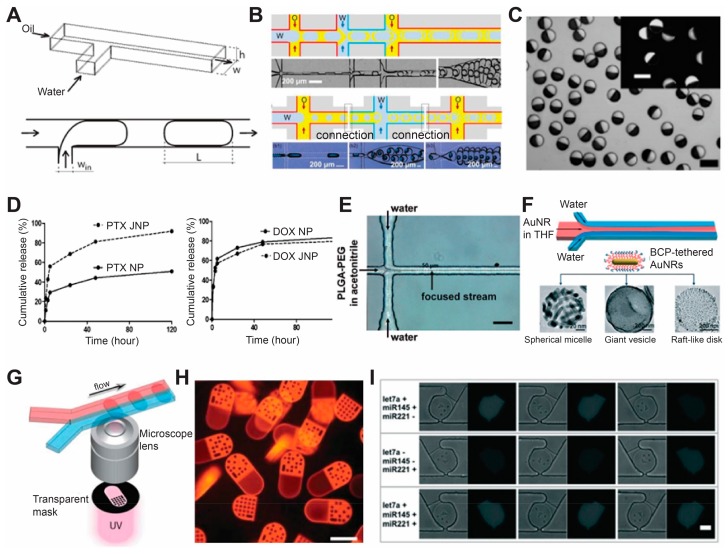
Microfluidic fabrication of nano/microparticles and their applications in drug delivery and disease diagnosis. (**A**) Single emulsion (**B**) multiple emulsion methods of droplet production in microfluidic channels [141], with copyright permission from © Royal Society of Chemistry; (**C**) Optical microscopy image of Janus particles (inset shows fluorescence image) [144], with copyright permission from © American Chemical Society; (**D**) Cumulative release of paclitaxel (left) and doxorubicin from Janus particle for co-delivery of two drugs [145], with copyright permission from © American Chemical Society; (**E**) A 2-dimensional HFF microfluidic device for generation of PLGA-PEG nanoparticles [147], with copyright permission from © American Chemical Society; (**F**) Hydrodynamic self-assembly of amphiphilic nanoparticles tethered with block copolymers [147], with copyright permission from © John Wiley and Sons; (**G**) Scheme of barcoded particle synthesis using flow lithography [145], with copyright permission from © The American Association for the Advancement of Science; (**H**) Fluorescence microscopy image of barcoded particles for multiplex analysis [145], with copyright permission from © The American Association for the Advancement of Science; (**I**) Multiplexed miRNA assay for three different dysregulation patterns using barcoded microparticles produced from the method in (**G**) [146], with copyright permission from © Royal Society of Chemistry. Scale bar 100 µm for (**C**,**H**), 50 μm for (**E**,**I**).

Advanced manufacturing techniques also allow production of micro/nano particles through microfluidic technology. It worth mentioning that the production of microfluidic devices strongly relies on advanced manufacturing techniques such as photolithography. In microfluidic channels, a small and precise amount of materials flow in micrometer-sized channel, and micro/nano particles with controllable sizes, shapes and hierarchy structures were created by polymerization of the liquid precursor via physical or chemical interaction with the surrounding fluid in the microchannels. (Figure 7A,B) [139,140]. Briefly, depending on the number of liquid phases employed in the fluidic devices, existing microfluidic techniques for producing micro/nano structures with controllable features can be classified into two groups: oil-in-water emulsion (or multi-phase fabrication) and one-phase fabrication (or known as flow photolithography). For the oil-in-water emulsion, two or more streams of immiscible liquid precursors flow in separate channels, and these liquid precursors were brought together to form emulsion droplets or by using hydrodynamic flow focusing techniques such as T-junction or nozzle confinement. [139,140]. Single, double and multiple emulsion droplets can be created via different design of microchannels (Figure 6A,B) [139,140]. These micro- or nano-scopic droplets will further undergo chemical or photo crosslinking to condense into solid particles. [139,140].

An extension of the above-mentioned microfluidic-based technique is the production of Janus particles through combination of two droplets from two parallel flow. (Figure 7C) [141]. Two drugs that are immiscible with each other (such as hydrophobic and hydrophilic) can be encapsulated and co-delivered in the same drug carrier (Figure 7D) [142]. Besides the droplet method, hydrodynamic flow focusing (HFF) is also applied in the microfluidic channel to produce nano/microparticles. Normally HFF is achieved by adding two addition inlets for generating sheath liquid and compressing the precursor solution to the center (Figure 7E,F) [143,144]. The abovementioned techniques can generally produce spherical micro/nano particle, while in most cases they lack the ability to produce non-spherical particles. The one-phase technique, developed by the Doyle group at MIT, can integrate microscope projection lithography into the production: photo-curable materials flow in the micro-channels, and a UV light passes through a photomask to crosslink the materials into micro/nano structures whose shape and size are defined by the photomask [145]. Moreover, this technique allows the generation of spatially encoded particles for fast barcoding of each individual particle. (Figure 7G,H) [145]. Thus far, therapeutic agents such as DNA, protein and microRNA probes have been encoded into the micro/nanostructures produced by this technique, allowing the micro/nano materials created by this technique to be used for biomedical applications such as drug delivery, cell tracking and disease diagnosis. (Figure 7I) [146].

Beyond all the above-mentioned techniques, other techniques have emerged for generating nanomaterials with well-controlled features [147,148]. Among these newly emerged fields, nanoarchitectonics are very promising in designing novel nanoparticles for therapeutic delivery. Nanoarchitectonics is a methodology that allows arranging nanomaterials by integrating several processes including atom/molecule manipulation, chemistry nanomanipulation or controlling materials with field or self-assembly [149]. This newly emerged method resembles the way that biological systems construct materials, thus this is a powerful bioinspired technique that could be employed to produce new micro/nanoparticles. The method has been employed to produce nanomaterials composed of small amphiphilic molecules, polymer micelles and molecular conjugates, which have been utilized in biomedical applications such as drug delivery. A thorough review of this field could be found in the existing literature [147,148,149]. 

## 4. Conclusions

In this wok, we reviewed the use of advanced manufacturing techniques for producing micro/nanoparticles with controllable features, as well as the use of these nanoparticles for drug delivery and bio-imaging applications. To give a detailed introduction of the field, we started this review by discussing the roles of micro/nanoparticles features (i.e., size, shape, component materials and surface property) in affecting their interactions with cells and in vivo. We then reviewed the major advanced manufacturing techniques that have been used to generate micro/nanoparticles with controllable features, emphasizing the use of these micro/nanoparticles for drug delivery and bio-imaging. The above work illustrated that advanced manufacturing techniques could be employed to produce micro/nanoparticles with controllable features that can be used to explore unique biomedical applications, especially drug delivery and cell tracking.
